# Supplementary material for: On-chip spectrometers using stratified waveguide filters
Source: Nat Commun. 2021 May 11;12:2704. doi: 10.1038/s41467-021-23001-6 (PMC8113243; doi:10.1038/s41467-021-23001-6)
Supplement: Supplementary file 1 — Supplementary Information [file 41467_2021_23001_MOESM1_ESM.pdf]

Supplementary Information for

“On-chip spectrometers using stratified waveguides filters”

Li et al.

Supplementary Note 1: Reconstruction Algorithm Comparison

Our spectrometer belongs to the type of devices that aim at recovering spectra with incomplete or even imperfect measurements. This area has been a hot research topic in the past decades. The fundamental problem is to solve the following equation:

$$minimize \ \|D - SP\|^2 \ subject \ to \ 0 \leq P \leq 1 \quad (1)$$

where D is a Nx1 matrix containing N independent measurement results from projecting the unknown input signal spectrum, I on the M-SWF filters. S is a NxM matrix constructed of the sampling basis, i. e., SWFs as N-rows and P is an Mx1 matrix containing M unknown values that needs to be estimated to correspond to the input signal spectrum. This problem is typically called underdetermined problem due to the condition N<<M. It has been proven that this problem can be solved with high confidence using advanced algorithms when the sampling matrix S is well designed(1, 2). Numerous algorithms have been developed to solve this problem. In this work, we chose CVX algorithm implemented in a MATLAB environment, which is a mature package developed for disciplined convex programming (3) and have been commonly used for similar problems(4). In the supplementary material we show the importance of using advanced algorithm dedicated for underdetermined problem as well as show the importance of including regularization when reconstructing the spectrum, especially when it contains broad spectral components.

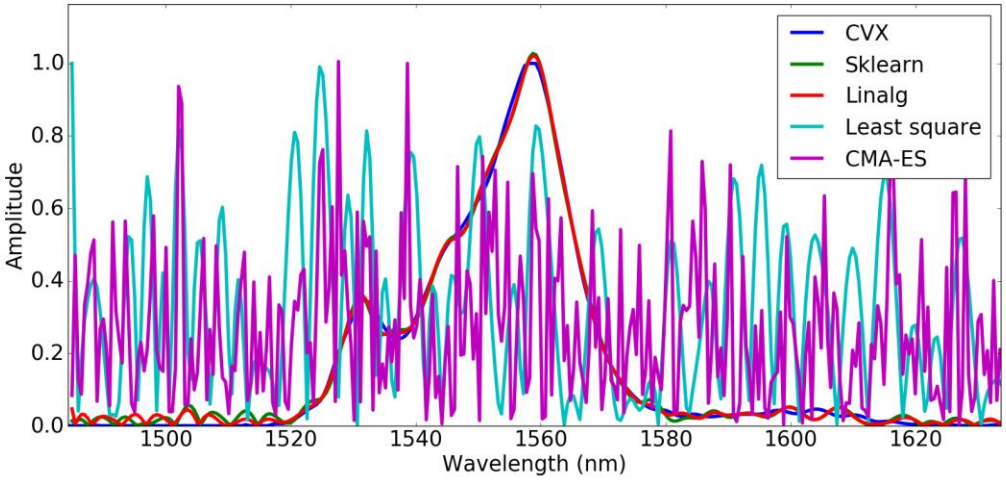

Supplementary Figure 1 Comparison of different reconstruction algorithms for input signal spectrum reconstruction

First of all, we show the results using a couple of different algorithms, including (i) CVX in MATLAB; (ii) machine learning (Sklearn)(5) and Linear algebra (Numpy.Linalg)(6) in Python; (iii) simple linear least square fitting approach; and (iv) a covariance matrix adaptation evolution strategy (CMA-ES) - a more complex algorithm aiming at finding global optimization for nonlinear problems(7). As it is clearly shown in Supplementary Figure 1, only CVX, Sklearn and Linalg can successfully reconstruct the spectrum, while the other optimization based approaches fail.

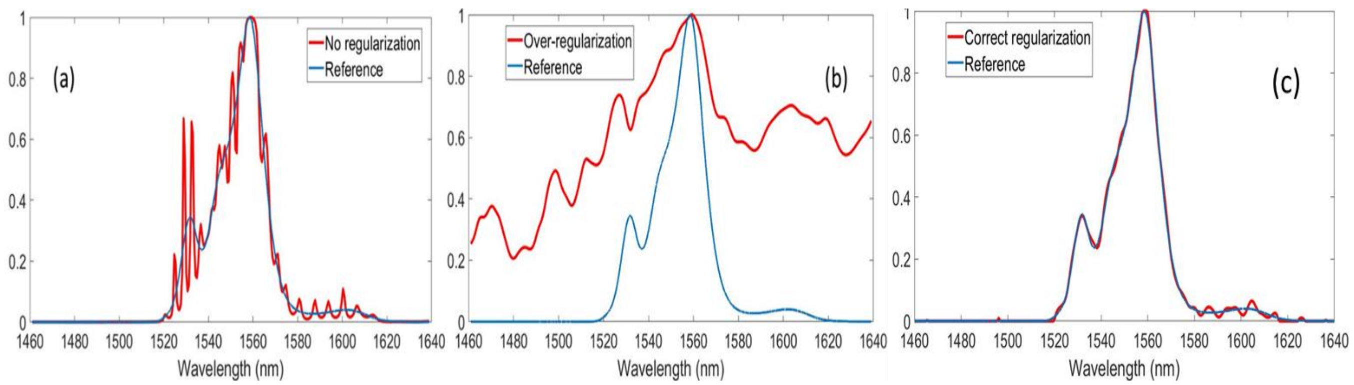

Supplementary Figure 2 The importance of choosing correct regularization weight. (a) shows the reconstruction without any regularization, random spikes appear. (b) shows the result when regularization weight is too large. And (c) presents the results with correct regularization coefficient.

Next we show the importance of regularization: As explained in the manuscript, it is necessary to include regularization in order to smoothen the reconstructed spectrum, otherwise the reconstructed spectrum exhibits random spikes as evident in Supplementary Figure 2(a). The regularization coefficient needs to be carefully chosen otherwise the performance will be poor when it is "over-regularized" (the regularization weight is too large) as also evident in Supplementary Figure 2(b). Also we plot the results using correct regularization weight in Supplementary Figure 2c). The correct regularization coefficient depends on the filters used for spectrum reconstruction, and it can be pre-determined by sending some known spectrum to the filters and adjusting the weight to get a good reconstruction result.

Supplementary Note 2: Analysis of the tolerance of the spectrometer regarding imbalance among different filter channels

Ideally, each port of the splitter should extract similar amount of power so that the individual filter can sample the incident signal with similar weight. To understand the impacts of the imbalance between different ports, we perform additional measurements by intentionally introducing extra coupling loss in evaluating some channels of the spectrometer. First measurement is to sweep the number of ports that suffer from a fixed amount of loss, which is 10dB, to see how the performance varies with increasing number of channels that has this additional loss. We see little impacts as evident in Supplementary Figure 3, mainly in the spectral components with low amplitudes.

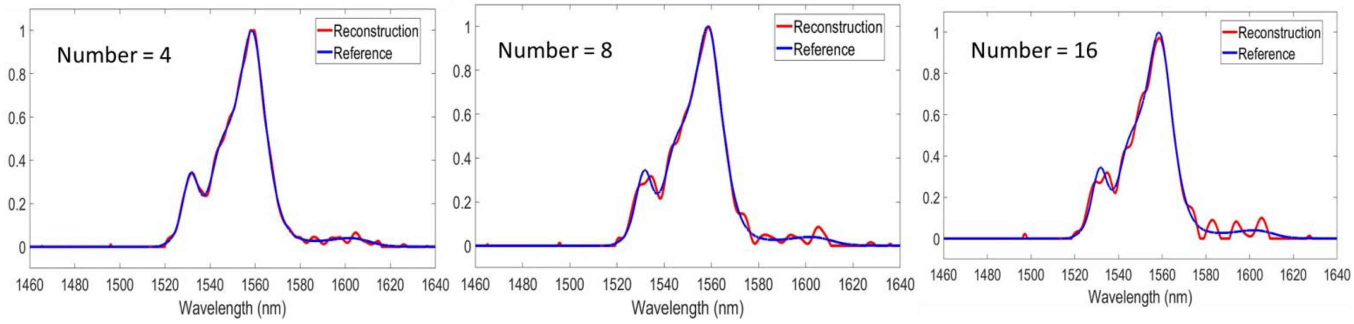

Supplementary Figure 3 Spectrometer performance with varying number of channels that exhibit extra 10dB loss.

Next, we fix the number of channels that exhibit extra loss to be 16 (half of the total channels) and sweep the magnitude of the loss to see the impacts. As plotted in Supplementary Figure 4, we could clearly observe performance degradation due to larger additional loss, namely, imbalance between channels. When the imbalance is about 14dB, we notice significant mismatch between the reconstructed spectrum and the reference measured by a commercial OSA. Therefore, we could conclude that the spectrometer is robust to imbalance between the channels. The reason is attributed to the rich features in the transmission spectra of the SWFs, that the extinction among different wavelengths can be over 10dB. So any imbalance that is smaller than the extinction in the filters' spectrum will not significantly affect the sampling of the signal.

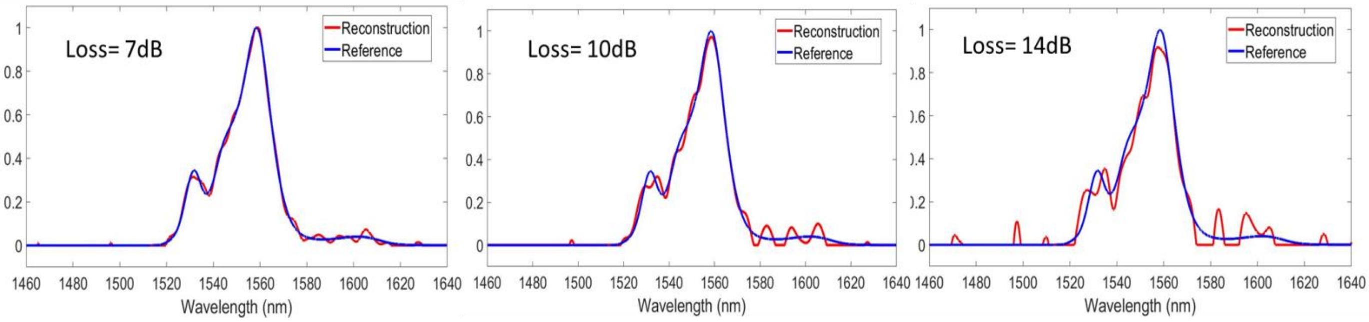

Supplementary Figure 4 How the magnitude of imbalance between channels will impact the spectrum reconstruction.

Supplementary Note 3: Analysis of the impacts of filters transmission spectra on spectrometer performance

There have been some demonstrations of single-shot spectrometers that use a set of broadband filters for sampling the incident signal(4, 8, 9). The numbers of filters used vary from 36 to over 360 and it is always claimed that simply using more filters can deliver better performance in terms of spectral resolution. So this raises two questions that have been neglected so far: how to determine the proper number of filters for spectrum reconstruction and is it true that using more filters can always lead to better performance. Ideally, we'd like to keep the number of filters low to keep high SNR and small footprint of the overall spectrometer. And we'd like to show here that, the minimum number of filters used for accurate spectrum reconstruction strongly depends on the filters' transmission spectra. Also, using more filters with similar transmission features cannot improve the resolution indefinitely, as the filters transmission spectra impose a fundamental limit on the resolution of the spectrometer.

Good filters for this type of single-shot spectrometer need to meet two requirements:

1. Each filter's transmission spectrum needs to be very different with others (low cross-correlation). Ideally, they should be orthogonal and the cross-correlation is 0 at each point. Therefore, the sampling of the incident signal using these filters generate linearly independent results. Then a small number of filters is needed to reconstruct unknown spectrum. While on the other hand, if filters' transmission spectra exhibit a significant degree of similarity, a large number of filters are required for good reconstruction of the spectrum. To verify this, we manually generate two sets of filters' spectra, the first set shows similarity in their transmission spectra as evident in Supplementary Figure 5(a), while the second set are more random and less correlated as plotted in Supplementary Figure 5(b). We use both sets to reconstruct an exemplar spectrum consisting of two broad peaks at 1540nm and 1560nm, respectively. For first set, the reconstruction using 30 filters shows poor quality, and 90 filters have to be used for good reconstruction as evident in

Supplementary Figure 5(c). While for second set, by using only 30 filters, a good reconstruction is generated as evident in Supplementary Figure 5(d). This explains why the number of filters used in previous demonstrations vary significantly(4, 8, 9).

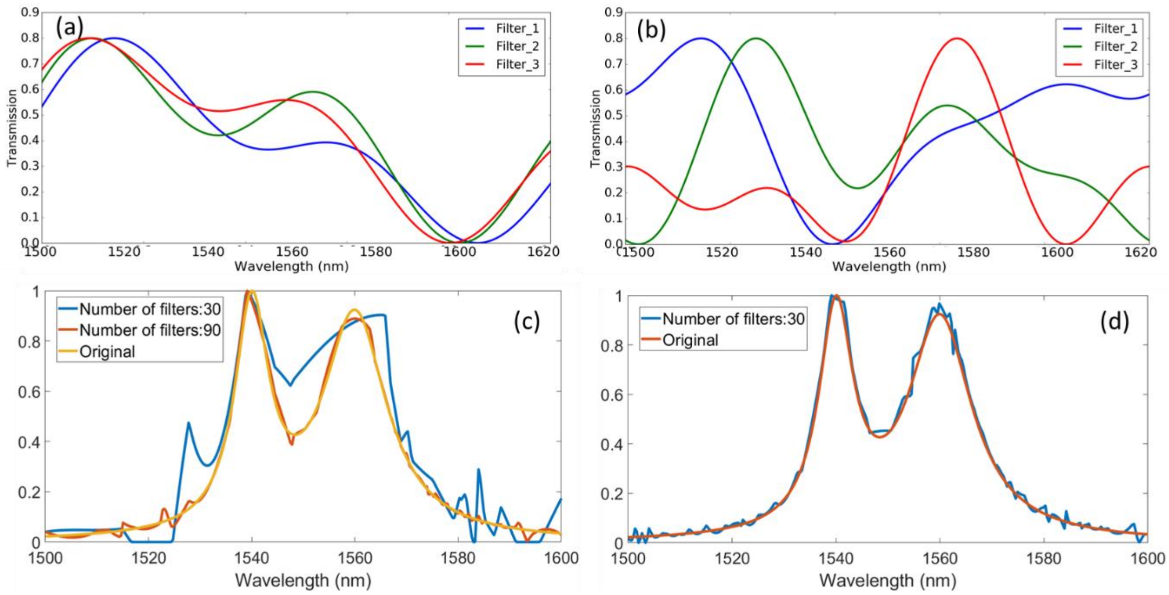

Supplementary Figure 5 Simulations showing how the similarity in the filters' transmission spectra will influence the spectrometer performance. (a) and (b) show two sets of filters for sampling the unknown spectrum. The set in (a) has similarity in individual filter's spectrum, while the set in (b) has very different filter spectra. (c) and (d) presents their reconstruction of a spectrum containing two broad peaks at 1540nm and 1560nm. For first set, using 30 filters shows poor reconstruction quality (c). While for second set, only using 30 filters can give a good reconstruction.

2. Individual filter's transmission spectrum needs to be sharp and random, in order to deliver high reconstruction resolution. In other words, it should have more distinguishable transmissions at two closely separated wavelengths, $|F(\lambda_1) - F(\lambda_1 + \Delta\lambda)| \gg 0$, where $F(\lambda_1)$ refers to the transmission at λ_1. For filters with smooth transmission spectra, increasing the number of filters used will not help to increase the resolution indefinitely as there is a fundamental limit imposed by the filters' transmission spectra. To clarify this point, we manually generate two sets of filters' spectra, the first set shows smooth features as shown in Supplementary Figure 6(a), while the second set are more random and sharper as plotted in Supplementary Figure 6(b). Next, we use both sets of filters for sampling a spectrum containing two narrow peaks at 1540nm (FWHM=0.8nm) and 1560nm (FWHM=1.2nm). The results are plotted in Supplementary Figure 6(c) and (d). Clearly, for the first set, increasing the number of filters from 5 to 20 indeed shows improvement in performance. But increasing from 20 to 50 doesn't help further increase the performance, the resolution is limited to about 7nm, due to smooth filters spectra. While for the set of filters with sharp features, both narrow peaks can be accurately reconstructed.

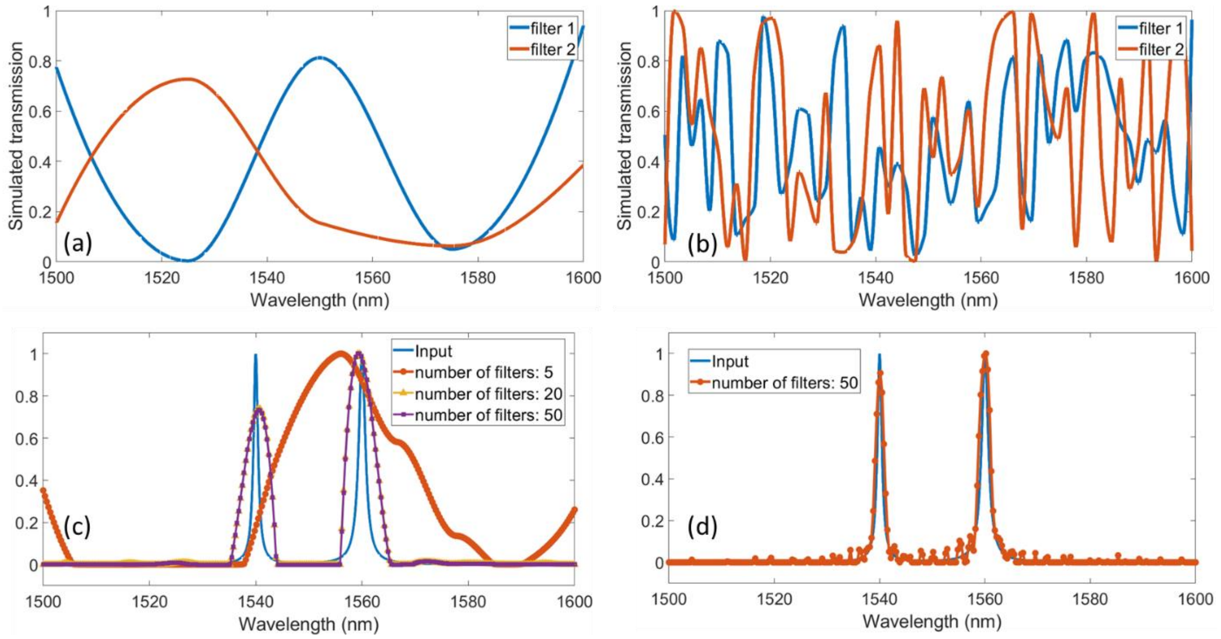

Supplementary Figure 6 Simulations showing how the sharpness in the filters' transmission spectra will influence the spectrometer performance. (a) and (b) show two sets of filters for sampling the unknown spectrum. The set in (a) has smooth spectra, while the set in (b) has very sharp features in their spectra. (c) and (d) presents their reconstruction of a spectrum containing two narrow peaks at 1540nm (FWHM=0.8nm) and 1560nm (FWHM=1.2nm). For first set, increasing the number of filters from 5 to 20 indeed shows improvement in performance. But increasing from 20 to 50 doesn't help further increase the performance, The resolution is limited to about 7nm, due to smooth filters spectra. While for the set of filters with sharp features, both narrow peaks can be accurately reconstructed.

In summary, the filters transmission spectra are more vital in determining the spectrometer performance. Increasing the number of filters used cannot improve the performance indefinitely.

Supplementary Note 4: Procedure to determine the correct regularization coefficient

The regularization coefficient is critical to the performance of the spectrometer. As explained in the main context, the coefficients for broad and narrow spectral components can be adjusted by sending known spectra to the spectrometer for reconstruction until good matchings are observed.

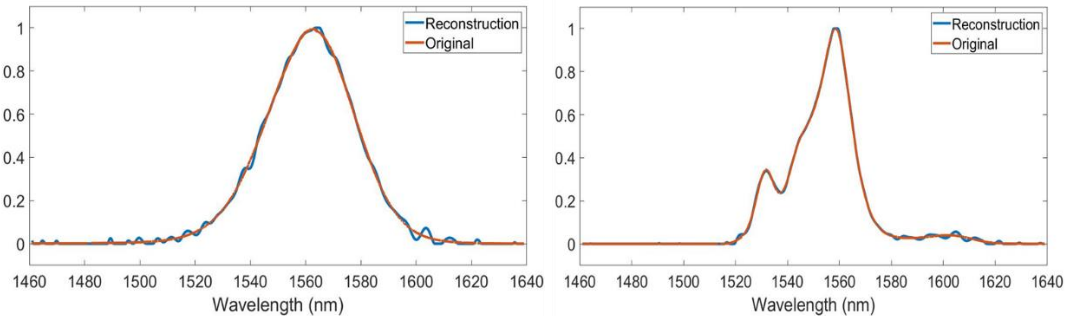

Supplementary Figure 7 Calibration and reconstruction using different spectra. Left panel shows the calibration using a broadband SLD source with quasi-Gaussian spectrum covering about 80nm span around 1560nm. With the adjusted regularization coefficient from the calibration procedure, the C+L band ASE spectrum can be reconstructed with high quality (right panel).

We use a broadband superluminescent laser diode (SLD) from Thorlabs with a quasi-Gaussian spectral profile that has 50nm 3dB bandwidth around 1560nm and use it as the calibration spectrum for broadband regularization coefficient. Then we use the calibrated regularization coefficient to reconstruct the broadband C+L ASE spectrum. The reconstructed spectra of both SLD used for calibration and ASE spectra used for testing are plotted in Supplementary Figure 7. It clearly shows that using the calibrated regularization coefficient obtained from SDL spectra allows accurate reconstruction of the broadband ASE spectrum

While for the narrowband regularization coefficient, we use the same tunable light source for both calibration and reconstruction due to limited choice of equipment. We used 10 spectra with narrow peaks at different locations for calibration and get 10 different values for regularization coefficients. Then the average of these 10 values of the regularization coefficients is set as the common regularization coefficient for narrowband spectrum, and perform spectra reconstruction containing unknown narrowband spectrum components.

Supplementary References:

1. Candes EJ, Romberg JK, Tao T. Stable signal recovery from incomplete and inaccurate measurements. Communications on Pure and Applied Mathematics: A Journal Issued by the Courant Institute of Mathematical Sciences. 2006;59(8):1207-23.
2. Donoho DL. Compressed sensing. IEEE Transactions on information theory. 2006;52(4):1289-306.
3. Grant M, Boyd S, Ye Y. CVX: Matlab software for disciplined convex programming. 2008.
4. Wang Z, Yi S, Chen A, Zhou M, Luk TS, James A, et al. Single-shot on-chip spectral sensors based on photonic crystal slabs. Nature communications. 2019;10(1):1020.
5. Komer B, Bergstra J, Eliasmith C, editors. Hyperopt-sklearn: automatic hyperparameter configuration for scikit-learn. ICML workshop on AutoML; 2014: Citeseer.
6. Heck A. Linear Algebra: The linalg Package. Introduction to Maple: Springer; 1996. p. 575-99.
7. Hansen N. The CMA evolution strategy: a comparing review. Towards a new evolutionary computation: Springer; 2006. p. 75-102.
8. Bao J, Bawendi MG. A colloidal quantum dot spectrometer. Nature. 2015;523(7558):67-70.
9. Zhu X, Bian L, Fu H, Wang L, Zou B, Dai Q, et al. Broadband perovskite quantum dot spectrometer beyond human visual resolution. Light: Science & Applications. 2020;9(1):1-9.
